# Reduction of astrogliosis and microgliosis by cerebrospinal fluid shunting in experimental hydrocephalus

**DOI:** 10.1186/1743-8454-4-5

**Published:** 2007-06-07

**Authors:** Janet M Miller, James P McAllister II

**Affiliations:** 1 Department of Neurosurgery, Wayne State University, Detroit, Michigan, USA; 2 Department of Physiology, Wayne State University, Detroit, Michigan, USA; 3 Department of Anatomy and Cell Biology, Wayne State University, Detroit Michigan, USA; 4 Department of Pediatric Neurosurgery, Children's Hospital of Michigan, Detroit, Michigan USA

## Abstract

**Background:**

Reactive gliosis has the potential to alter biomechanical properties of the brain, impede neuronal regeneration and affect plasticity. Determining the onset and progression of reactive astrogliosis and microgliosis due to hydrocephalus is important for designing better clinical treatments.

**Methods:**

Reactive astrogliosis and microgliosis were evaluated as the severity of hydrocephalus increased with age in hydrocephalic H-Tx rats and control littermates. Previous studies have suggested that gliosis may persist after short-term drainage (shunt treatment) of the cerebrospinal fluid. Therefore shunts were placed in 15d hydrocephalic rats that were sacrificed after 6d (21d of age) or after 21d (36d of age). Tissue was processed for Western blot procedures and immunohistochemistry, and probed for the astrocytic protein, Glial Fibrillary Acidic Protein (GFAP) and for microglial protein, Isolectin B4 (ILB4).

**Results:**

In the parietal cortex of untreated hydrocephalic animals, GFAP levels increased significantly at 5d and at 12d compared to age-matched control rats. There was a continued increase in GFAP levels over control at 21d and at 36d. Shunting prevented some of the increase in GFAP levels in the parietal cortex. In the occipital cortex of untreated hydrocephalic animals, there was a significant increase over control in levels of GFAP at 5d. This trend continued in the 12d animals, although not significantly. Significant increases in GFAP levels were present in 21d and in 36d animals. Shunting significantly reduced GFAP levels in the 36d shunted group. Quantitative grading of immuno-stained sections showed similar changes in GFAP stained astrocytes.

Immuno-stained microglia were altered in shape in hydrocephalic animals. At 5d and 12d, they appeared to be developmentally delayed with a lack of processes. Older 21d and 36d hydrocephalic animals exhibited the characteristics of activated microglia, with thicker processes and enlarged cell bodies. Following shunting, fewer activated microglia were present.

Histologic examination of the periventricular area and the periaqueductal area showed similar findings with the 21d and 36d animals having increased populations of both astrocytes and microglia which were reduced following shunting with a more dramatic reduction in the long term shunted animals.

**Conclusion:**

Overall, these results suggest that reactive astrocytosis and microgliosis are associated with progressive untreated ventriculomegaly, but that shunt treatment can reduce the gliosis occurring with hydrocephalus.

## Background

Reactive astrogliosis and microgliosis is a common occurrence in hydrocephalus [1-4], and reducing the presence of excess reactive glial cells is important for the brain tissue to function normally. There is no cure for hydrocephalus, and shunting is merely a palliative treatment. Therefore, we believe it will be beneficial to determine the time of onset of reactive astrogliosis and microgliosis due to hydrocephalus. We also believe that determining the reversibility of this type of gliosis is important for devising the most appropriate treatment. The function of resting astrocytes and microglia is to aid cellular growth and development. Upon activation by injury, these glial cells release cytokines and chemokines which aid in the recruitment of other astrocytes and microglia to the site [5]. This recruitment can lead to the formation of a glial "scar", which has the potential to block the growth of new neuronal processes, and also may impede neo-vascularization, thus inhibiting recovery after injury [5-7]. Although observations of reactive astrocytes and microglia have been characterized in hydrocephalus by histological and quantitative studies, the temporal progression, severity, reversibility, and the specific cellular elements involved, is not known [1-3,8-11]. Therefore, a clear understanding of the mechanisms involved in the genesis and progression of hydrocephalus is important for improving diagnostic and therapeutic options.

Congenital hydrocephalus is a condition usually marked by an excessive accumulation of cerebrospinal fluid (CSF) within the cerebral ventricles resulting in ventricular enlargement. This condition affects between 0.48 to 0.81 infants per 1000 live births [12,13], and up to 78% of patients suffer persistent deficits after treatment, possibly due to reactive astrogliosis and microgliosis [13-19]. Our previous studies have shown that the RNA level of Glial Fibrillary Acidic Protein (GFAP) specific for astrocytes, increases with the progression of hydrocephalus in both a congenital model of rodent hydrocephalus (H-Tx rat) and a kaolin model of induced hydrocephalus in kittens [20]. Additionally, Mangano *et al *[21] illustrated that microglial cell proliferation and activation increased in regions of the sensorimotor cortex and auditory cortex during the progression of hydrocephalus in moderately affected H-Tx rats. Furthermore, Yoshida *et al *found that GFAP labeled reactive astrocytes were present surrounding cystic lesions in severely hydrocephalic H-Tx animals, but they were not able to detect a significant increase in GFAP labeled astrocytes in the white matter surrounding the ventricles [11,22]. Clinically, increased levels of GFAP have been found in the CSF of patients with normal pressure hydrocephalus, and in patients who developed secondary hydrocephalus due to subarachnoid hemorrhage [23-26], and the possibility of using GFAP levels as a diagnostic tool for hydrocephalus is currently being explored [27,28]. Although these studies contribute to the recognition that gliosis exists in hydrocephalus, they fail to determine the timing of the glial activation. Therefore, in the present study utilizing H-Tx rats, the temporal progression of reactive astroglia and microglia was examined in two areas of the cerebral cortex severely affected by the development of hydrocephalus, the parietal and occipital cortex.

In addition, an important question with clinical impact still remains: can excessive reactive astrogliosis and microgliosis be reduced or prevented by CSF drainage? Previous studies in a kaolin-induced kitten model of hydrocephalus demonstrated that shunting reduced the amount of GFAP present, but the results were quite variable, and GFAP levels began to rise over time [20]. The unique value of the present study is that it characterizes, in a clinically relevant model of hydrocephalus, the effect of both long term and short term shunting on reactive astrocytosis and microgliosis. The present study aims not only to identify the temporal progression and location of astrogliosis and microgliosis, but also to determine if the gliotic response is reversible or prevented by CSF drainage. Our hypothesis is that reactive gliosis (specifically reactive astrogliosis and microgliosis) closely coincides with the onset of neonatal hydrocephalus and that this gliosis will be prevented or reversed by CSF drainage through shunting.

## Methods

### Experimental design

All animal experiments were approved by the Wayne State University Institutional Review Board and were conducted following the National Institute of Health Guide for the Care and Use of Laboratory Animals (NIH Publication No. 80-23, revised 1996). The H-Tx rats (H-Tx/hcj strain) originated from Dr. Hazel Jones, University of Florida. Animals were maintained on 12 h dark-light cycles in a controlled environment with free to access food and water at all times.

Brains from hydrocephalic H-Tx rats were examined utilizing immunohistochemistry and immunoblotting at the following postnatal ages: 5d, 12d, 21d and 36d, and compared to non-hydrocephalic age-matched control H-Tx rats (n = 5 for each group). The effect of CSF drainage was examined by inserting a shunt into severely hydrocephalic animals at 15d, and examining them after 6d (21d of age) or after 21d (36d of age). These shunted animals were compared to age-matched un-treated hydrocephalic H-Tx rats and their respective control littermates (n = 5 for each group).

### Hydrocephalus model

In this study, the H-Tx rat model of congenital hydrocephalus was utilized. Although this is not an exact replica of the human condition, we believe this model appropriately mimics congenital human hydrocephalus. H-Tx animals have a slowly progressive form of hydrocephalus which is primarily caused by an alteration of the cerebral aqueduct occurring between embryonic day 18 and post-natal day 5 [29-36]. Because the cranial sutures of these young animals are not yet fully fused, the skull is expandable and accommodates the rising ventricular volume, thus allowing for overt visual identification of the hydrocephalic individuals.

### CSF diversion (shunt treatment)

To examine the effects of shunting, Teflon-coated catheters coupled to Heyer-Schulte low-pressure neonatal valves (Heyer-Schulte- Integra, New Jersey, USA) were inserted into the lateral ventricle of hydrocephalic H-Tx rats at 15d. This age approximates the stage when hydrocephalus in these animals advances from a moderate to a severe state, and the developing rat cerebral cortex is close to that of a newborn human [37]. Animals were given a pre-operative oral dose of Cephalexin antibiotic (50 mg/kg) and the same dose was given twice daily for 5d postoperatively to help prevent infection. Animals were anesthetized with 2% halothane and prepared for sterile surgery, and all procedures were performed under aseptic conditions. A small incision was made over the skull and over the lower back of the animal. A small burr hole was created in the skull 1 mm lateral to the midline and 2 mm posterior to Bregma. After piercing the dura mater, the tip of the shunt catheter was advanced without use of a stylet into the lateral ventricle, and fixed to the skull using one to two drops of ethyl cyanoacrylate (Krazy^©^) glue. The distal end of the catheter was left lying in the subcutaneous tissue above the distal lumbar vertebrae of the spine near the caudal vertebrae. This allowed for movement of the distal end of the catheter during growth, and prevented the shunt catheter from being pulled out of the burr hole. The patency of the shunt was tested by withdrawing a few drops of CSF through the distal end of the catheter. Skin incisions were closed using tissue staples, and an ear tag was placed for identification purposes. The animal was removed from anesthesia, allowed to recover on a heated surface, and returned to the cage with its mother and littermates. In the days following shunt insertion, any animals displaying neurologic dysfunction or lethargy, suggesting a shunt failure or infection, were sacrificed and not utilized for this study.

### Sacrifice and fixation

Animals used for histology and immunohistochemistry were deeply anesthetized with (1–4 ml depending on age) 4% chloral hydrate i.p. and perfused transcardially with saline followed by 4% paraformaldehyde fixative in 0.1 M PBS pH 7.4, for optimal preservation of brain morphology. Following perfusion, the brain was removed from the skull, sectioned coronally into thirds (frontal, parietal and occipital sections) and placed into paraformaldehyde. The hydrocephalic animals were classified as having severe hydrocephalus through observation of the dilation of the ventricles and the thickness of the cortex, and only those animals with clearly defined hydrocephalus were included in this study. The control littermates were also verified visually by inspection of the ventricles. Following 2–4 h of post-fixation, the brain was removed from the fixative, rinsed and stored in 0.1 M PBS until paraffin embedding.

### Fresh tissue preparation

Separate groups of rats were used for Western blotting analyses and the tissue was prepared using a fresh frozen technique. Animals were deeply anesthetized by inhalation of a halothane from a soaked cotton swab, decapitated, and the brain rapidly removed and dissected on ice. Samples from the cerebral cortex were snap-frozen in liquid nitrogen and stored at -80°C. Brain tissue was homogenized in buffer containing: 50 mM NaF, 50 mM HEPES (4-(2-Hydroxyethyl) piperazine-1-ethanesulfonic acid) (pH 7.5), 2.5 mM 4-dithiotreitol-d_10 _(DTT), 120 mM KCl, 4 mM MgOAc, 1 mM ethylenediaminetetraacetic acid (EDTA), 20 mM glycerophosphate, 10 μg/ml pepstatin A, 10 μg/ml aprotinin, 10 μg/ml leupeptin, 1 mM orthovanidate, 250 nM okadaic acid and 1 mM phenylmethanesulfonyl fluoride (PMSF). Protein concentration was determined by the Lowry method. Samples were added to 2X loading buffer containing: 0.125 M Tris-HCl pH 6.8, 4% sodium dodecyl sulfate (SDS), 20% glycerol, and 10% β-mercaptoethanol and boiled for 90 sec. Equal protein amounts were loaded into lanes for electrophoresis procedures.

### Western blot analysis

Aliquots containing 50 μg protein from brain homogenates were electrophoresed in 10% SDS-polyacrylamide gels. To allow for comparison between membranes, a sample from one individual animal was loaded onto every gel, and all samples on the membrane resulting from that gel were standardized using the consistently loaded sample. Beta actin expression was also used frequently to ensure standard loading of gels.

Proteins were then electrophoretically transferred onto a nitrocellulose membrane, which was fixed in 10% acetic acid and 25% isopropanol for 15 min to ensure protein immobilization. The membrane was then placed at 4°C for 15 min, and rinsed 10 times in dH_2_O followed by one rinse in 50 mM Tris, pH 7.4, 200 mM NaCl. All subsequent procedures were performed at room temperature.

For detection of GFAP, non-specific antibody binding was pre-blocked in 5% low-fat dried milk dissolved in TTBS (100 mM Tris, pH 7.4, 0.9% NaCl, 0.1% v/v Tween-20) for 45 min with gentle agitation. After rinsing with TTBS, the membranes were incubated with anti-GFAP antibody 1:1,000 in TTBS (DAKO, USA) for 60 min and gently agitated at room temperature.

After incubation in primary antibody, membranes were washed 3X in TTBS, and incubated for 30 min in TTBS containing anti-rabbit IgG-horseradish peroxidase conjugate at a 1:10,000 dilution. The membranes were then washed 3X in TTBS, incubated in Enhanced Chemiluminsence (ECL – Amersham, USA) kit detection reagents for 90 sec, drained, covered with plastic wrap and contact-exposed to film. After film development, the bands were quantified by densitometry (Intelligent Quantifier, Bio Image Inc. version 3.0.0, Michigan, USA).

### Immunohistochemistry

All layers of the occipital cortex and the parietal cortex were examined from perfusion-fixed coronal sections with immunohistochemical techniques using antibodies specific for the astrocytic protein, GFAP, and microglial protein, ILB4. Additionally, the area surrounding the cerebral aqueduct was examined by the same immunohistochemical techniques. Labeled cells were identified using brightfield or fluorescent techniques using a Leica DMRE microscope (Leica Microsystem Products, New Jersey, USA).

Brains were embedded in paraffin and sectioned at 10 μm using standard histology procedures. Before staining, the slides were de-paraffinized and rehydrated, and subjected to antigen retrieval by placing mounted slides into 10 mM citrate buffer (pH 6.0) preheated to 90–100°C for 20 min, cooled for 20 min in buffer after removal from heat, and then washed in 0.1 M PBS.

For GFAP immunostaining, hydrated sections were incubated in 3% H_2_O_2 _for 10 min. The sections were then washed in dH_2_O and incubated in 5% goat serum diluted in 0.1 M PBS for 20 min and then in anti-GFAP antibody, 1:300 (DAKO, USA) diluted in an antibody diluent reagent solution (Zymed, California, USA). Primary antibody was then removed with a modified phosphate buffered saline (MPBS) containing 50 mM K_2_HPO_4_, 10 mM NaH_2_PO_4_, and 10 mM NaCl, and an anti-rabbit secondary antibody (1:200, Vector Laboratories, USA) was applied for 10 min. The secondary antibody was rinsed off with MPBS and an avidin-biotin complex (ABC kit, Vector Laboratories, USA) was applied for 30 min. Color was developed for 2–10 min using an un-enhanced DAB kit (Vector Laboratories, USA). Slides were dehydrated by passing them through increasing concentrations of alcohol and three changes (5 min each) of xylene, cover-slipped using Permount (Fisher Scientific, USA), a non-aqueous mounting media, and allowed to dry overnight. For fluorescence microscopy, the same primary GFAP antibody was used as described above, however, following primary incubation, tissue was incubated in a TRITC-labeled anti-rabbit secondary antibody (1:400, Sigma Aldrich, USA) used for 2 h at room temperature. Excess antibody was rinsed off and cover slips applied using Aquamount (Fisher Scientific, USA) or a DAPI fluorescent stain-impregnated hard-set mounting medium (Vector Laboratories, USA) to label nuclei.

ILB4 from *Griffonia simplicifolia *is a marker for brain microglia and peri-vascular cells [38]. Following rehydration, sections underwent antigen retrieval as described above, and were incubated in anti-ILB4 antibody 0.2 mg/ml (HRP labeled, Sigma Aldrich, USA) for 2 h at room temperature. The slides were washed with deionized water, and color developed with a nickel enhanced 3,3'-diaminobenzidine (DAB) kit for up to 10 min (Vector Laboratories, USA). The DAB solution was rinsed off, and the slides were dehydrated and cover slipped as above. For fluorescent staining, FITC-conjugated ILB4 (Sigma Aldrich, USA) primary antibody was used at 0.2 mg/ml and incubated at room temperature for 2 h. The slides were then rinsed and prepared for examination as described above.

### Analysis of data and statistics

Western blots were quantified by scanning densitometry and the data was analyzed using a Mann-Whitney U test for two groups, or a Kruskal-Wallis test for 3 groups, followed by individual Mann-Whitney U tests with a Bonferroni correction for between group comparison. For the quantitative assessment of astrocyte staining, the number of positively stained GFAP cells was graded on a four-point scale. A score of 1 indicated GFAP-labeled cells at a relatively low quantity, 2 and 3 indicated medium and moderate amounts, and 4 indicated that GFAP labeled cells were present in high abundance. The data was then analyzed using the Kruskal-Wallis and Mann-Whitney U tests followed by a Bonferroni correction as above.

**Figure 1:**
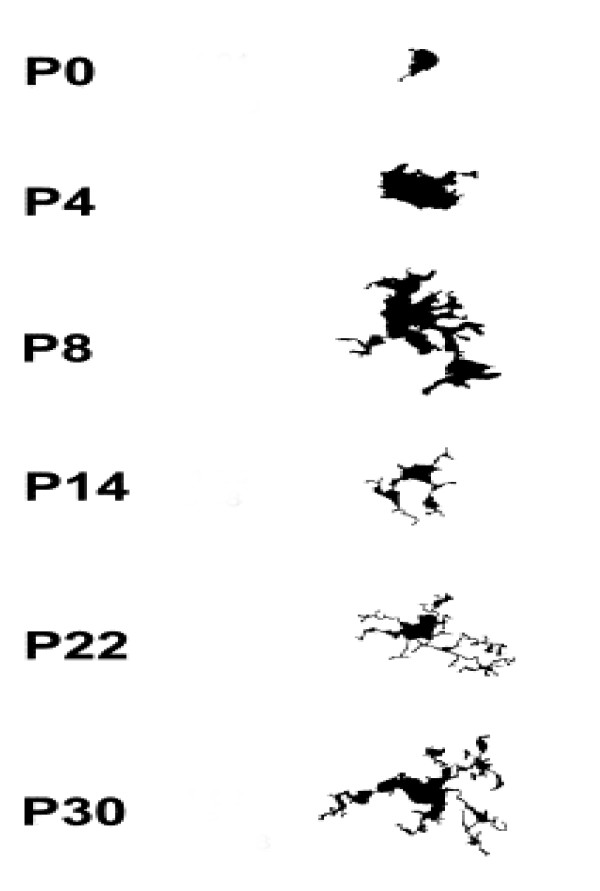
Ramification of microglia. This drawing illustrates the maturation or ramification of the microglial cell in the rat cortex (adapted from Orlowski *et al *[42]). P indicates postnatal day. Notable is the time it takes to develop fully ramified microglia.

ILB4 positive cells were analyzed from their morphologic appearance, which is a commonly accepted method to judge relative reactivity [21,39,40], rather than on the overall numeric density. Developmentally, microglia alter their morphology dramatically during the early postnatal period [41]. Microglia change from amoeboid-like cells at postnatal day 0 to completely ramified microglia over the first three weeks of post-natal development as seen in the figure adapted from Orlowski *et al *(Fig. 1) [39,41-43]. Due to the dramatic changes in normal cellular morphology observed during the first few weeks of life, each age group was treated individually, and was not directly compared to the other age groups. Therefore, for morphologic analysis of microglia in this study, hydrocephalic animals were compared only to their age-matched control and age-matched shunted counterparts.

## Results

### Gross observations: untreated hydrocephalus

**Figure 2:**
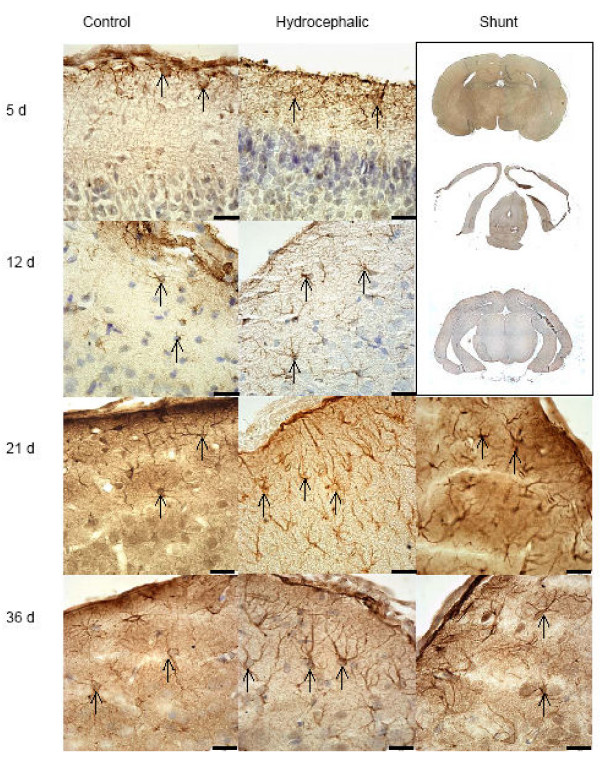
Histology of GFAP labeled astrocytes (cortical layers 1–3): GFAP containing cells are represented by brown staining (arrows). These images are representative of the areas of the cortex that were graded for with quantitative scaling. The hydrocephalic animals have more astrocytes in comparison to the age-matched control animals. Shunting reduced the relative overall appearance of stained astrocytes, although not to control levels. Scale bars = 25 μm. The upper right corner shows low power images of control (upper), untreated hydrocephalic (center) and shunted animal (lower) at 36d. Shunting resulted in re-expansion of the cortex, and dramatically reduced the apparent ventricular volume.

**Figure 3:**
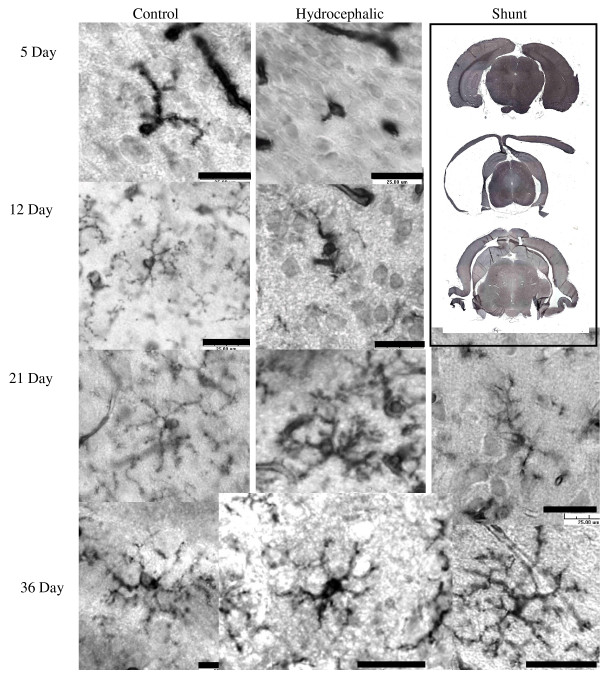
Isolectin B4 antibody staining for detection of microglia (in cortical layers 2–3). Microglial morphology was observed in the cortex of in control, hydrocephalic and shunted animals. In the 5d and 12d hydrocephalic animals, a relative lack of processes on the microglia cell was evident, while the 21d and 36d hydrocephalic animals, had shorter thicker processes than control. Following shunting in both age groups, a return of fine-branched processes was seen. Scale bar = 25 μm. Low power images of brains from 36d rats at the upper right demonstrate the gross effect of shunting (lower image) on cortical thickness and ventricular volume when compared to the control (upper) and hydrocephalic brain (center).

At sacrifice, all untreated hydrocephalic animals were easily identified by the presence of large, domed heads. Upon removal of the brain, these animals had a noticeable expansion of the lateral ventricles and thinning of the cortex and were classified as having severe hydrocephalus by visual inspection. This dramatic increase in ventricular volume and the thinning of the cortex is evident in low-power images (Figs. 2 and 3). All animals included in this study had similarly thinned cortices.

### Gross observations: treated hydrocephalus

At the time of sacrifice, both the short-term and long-term shunted animals had decreases in apparent ventricular size when compared to untreated hydrocephalic rats of the same age. Additionally, the cortex of the shunted groups was thicker when compared to untreated hydrocephalic animals, although the thickness did not appear to return to that of control levels. This reduction in ventricular volume and the increase in cortical thickness can be seen in low power images, which depict the brain of a shunted animal at 36d (Figs. 2 and 3).

### GFAP analysis by Western blots: untreated hydrocephalus

**Figure 4:**
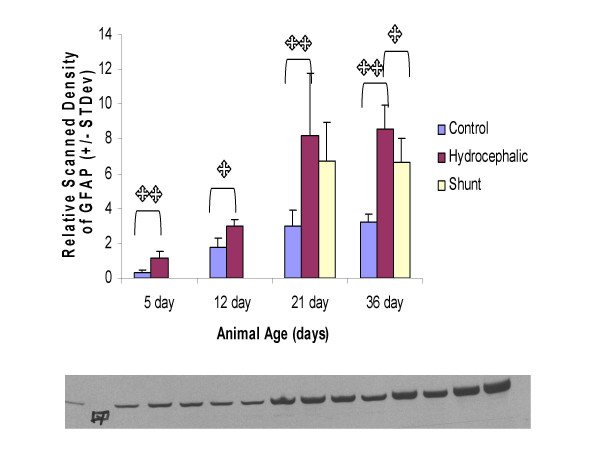
Scanning densitometry analysis of Western blots for GFAP in the parietal cortex. Above: Histogram of relative density. Overall, GFAP levels increased with age and with the progression of hydrocephalus, with significantly higher levels of GFAP being present in the 5d and 12d hydrocephalic animals. GFAP content also significantly increased over control in the 21d and 36d hydrocephalic animals. Shunting reduced GFAP levels from untreated hydrocephalic levels by 18% in the 21d shunted group (not significant). In the 36d group, there was a significant 23% decrease in the long term 36d shunted animals. Single asterisks indicates statistical significance at *p *< 0.05, double asterisk indicates significance at *p *< 0.01, brackets indicate comparison group. N = 5 for each group, values are means +/- standard deviation. Below: Immunoblot example showing clarity of bands, and standard in the left lane. The samples from the left are: lanes 3–7, 21 day control animal, 8–12, 21d shunted animals (shunted at 15d) and lanes 13–15, three of the 21d untreated hydrocephalic animals.

In the parietal cortex, GFAP levels in 5d hydrocephalic animals as measured by scanning densitometry of Western blots, were significantly increased by 3.68X compared to control animals (*p *< 0.01) (Fig. 4). In 12d hydrocephalic animals, the GFAP levels were also increased over controls by 1.69X (*p *< 0.05). The same trend in GFAP continued in the 21d hydrocephalic animals (2.77X; *p *< 0.01), and also in the 36d animals (2.69X; *p *< 0.01).

**Figure 5:**
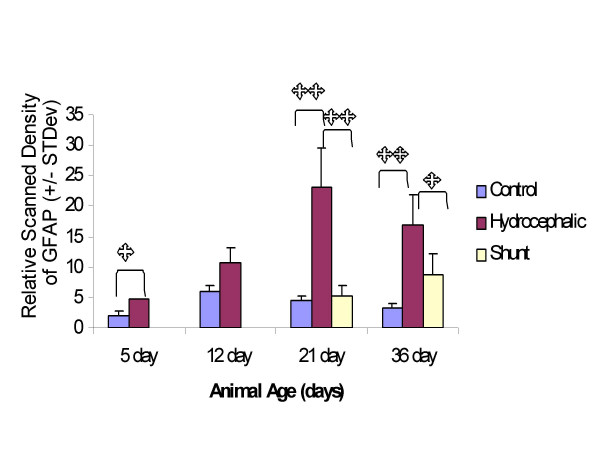
Scanning densitometry analysis of Western blots for GFAP in the occipital cortex: histogram of relative density. There was an increase in GFAP levels with hydrocephalus at all ages, although at 12d it was not significant. There was a significant increase over control at 5d, 21d and at 36d. In the shunted groups, GFAP was significantly reduced from untreated hydrocephalus by 76.9% at 21d and by 48.2% at 36d. Single asterisks indicates statistical significance at *p *< 0.05, double asterisk indicates significance at *p *< 0.01, brackets indicate comparison group. N = 5 for each group, values are means +/- standard deviation.

In the occipital cortex, there was a similar trend toward raised levels of GFAP in hydrocephalic animals compared to age-matched controls, although at 12d with a 1.81X increase the difference was not significant (*p *> 0.05). At the other ages, GFAP expression was increased over age-matched controls by 2.46X at 5d (*p *< 0.05,) by 5.26X at 21d (*p *< 0.01), and by 5.23X at 36d (*p *< 0.01) (Fig. 5).

### GFAP analysis by Western blots: treated hydrocephalus

GFAP levels in the parietal cortex exhibited significant alterations between control, hydrocephalic and shunted rats at 21d (*p *< 0.01). Shunt-treated animals had an 18% decrease in GFAP when compared to the untreated hydrocephalic animals, but this failed to reach significance (Fig. 4). At 36d, the shunted animals had a 23% decrease in GFAP levels when compared to the untreated 36d hydrocephalic animals (**p **< 0.05). An example of a representative Western blot is shown in Fig. 4.

The effect of shunting in the occipital cortex was more dramatic in reducing the levels of GFAP. Shunting hydrocephalic animals at 15d and allowing them to recover until 21d significantly reduced the amount of GFAP expression by 77% when compared to the untreated hydrocephalic animals (*p *< 0.01) (Fig. 5). This was similar to the expression level in control rats at 21d. Shunting at 15d and allowing a three-week post-shunt survival period until 36d, significantly reduced GFAP expression by 48.2% (*p *< 0.05).

### Astrocyte histology: untreated hydrocephalus

**Figure 6:**
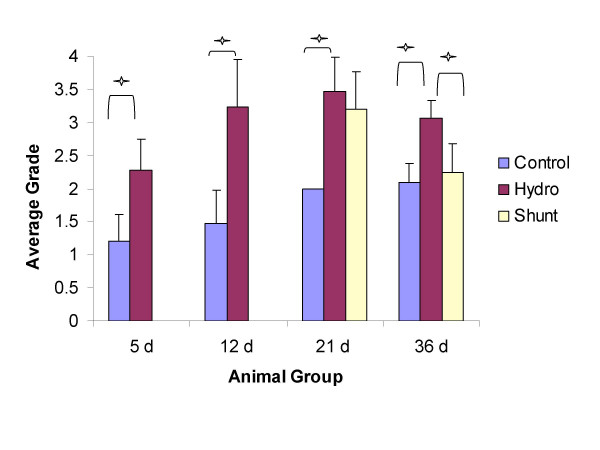
Histogram of histologic grading for relative abundance of GFAP labeled cells (cortical layers 1–4). There were significant increases in relative abundance of positively stained astrocytes in hydrocephalic rats at all age groups, similar to results from the Western blot experiments. Shunting reduced the abundance of stained cells in the 21d and in the 36d animals, but at 21d this was not significant. One asterisk indicates significance, *p *< 0.05, using a Mann-Whitney non-parametric test for two group comparisons or Kruskal-Wallis non-parametric test for three-group comparison (n = 5 for each group, values are means +/- standard deviation).

Qualitative histologic examination of tissue labeled with GFAP revealed astrocytes present throughout all layers of the cortex with relative increases in the number of positively stained cells found in untreated hydrocephalic animals, regardless of age (Fig. 2). Following quantitative grading based on the relative abundance of astrocytes in sections, the data was then compared and graphed (Fig. 6).

**Figure 7:**
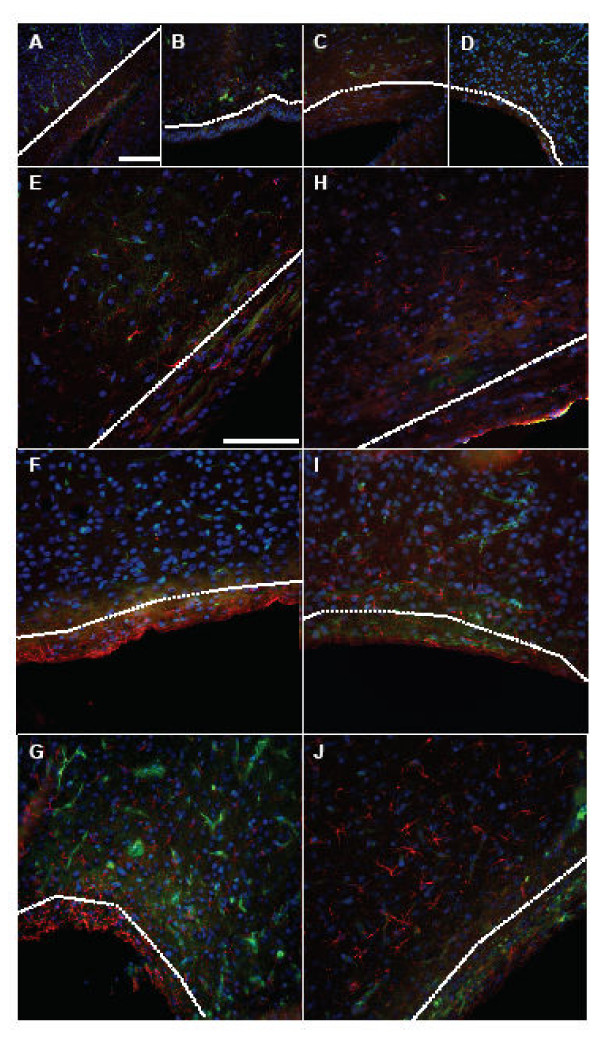
Fluorescent labeling of peri-ventricular white matter (from the parietal cortex region). Histologic sections were triple labeled to detect the presence of GFAP (red), ILB4 (green) and DAPI labeled nuclei (blue). No dramatic differences in relative abundance of staining were detectable between the 5d control (A) and hydrocephalic (B) animals, or between the 12d control (C) and hydrocephalic animals (D). There was increased abundance of GFAP staining in the peri-ventricular white matter of 21d hydrocephalic (F) animals when compared to their matched controls (E), this trend was also noticed between the 36d (I) hydrocephalic rats and their controls (H). Both 21d (G) and 36d (J) shunted rats had a slight increase in overall abundance of staining over control animals (E and H respectively). In the 36d shunted animals (J) GFAP positively labeled cells appeared to not be in a concentrated band as in the 21d shunted animals (G), but were present in a less dense band of staining, with cells migrating and distributed farther away from the peri-ventricular white matter. Microglia distribution in the 21d shunted animals (G) was slightly increased, while the 36d animals (J) did not show dramatic alterations in microglia. White dashed line demarcates cortical grey matter from peri-ventricular white matter. Scale bar = 100 μm.

**Figure 8:**
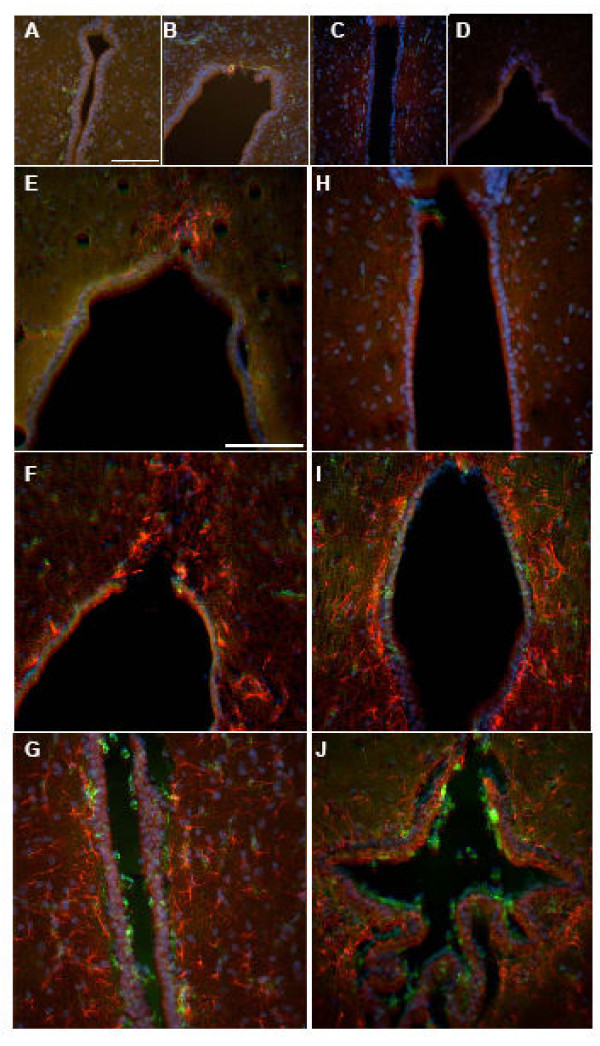
Fluorescent labeling of third ventricle region leading to the cerebral aqueduct. Histologic sections were triple labeled to detect the presence of GFAP (red), ILB4 (green) and DAPI (blue). In both the 5d control (A) and hydrocephalic (B) animals and 12d control (C) and hydrocephalic (D), there were no apparent differences in relative abundance of astrocytes or microglia cells. In the 21d animals, there was a remarkable increase in the abundance of astrocytes present in the hydrocephalic animals (F) when compared to the control animals (E). This dramatic increase in staining abundance was also noted between the control (H) and hydrocephalic (I) 36d animals. Shunting these two groups had the effect of reducing staining intensity and density of the astrocyte cells in the peri-aqueductal area of the untreated hydrocephalic animals. There was an increase in the microglial population in both of the shunted groups on the ventricular surface(G and J). Scale bar = 100 μm.

At 5d, upon visual examination, the control and hydrocephalic animals showed small numbers of GFAP labeled astrocytes, and these were found throughout all cortical layers (Fig. 2). Upon assigning a grade, the 5d hydrocephalic animals had a significant increase in the number of astrocytes present when compared to 5d non-hydrocephalic animals (2.3x, *p *< 0.05) (Fig. 6). When examining the periventricular white matter (Fig. 7A,B) or in the area surrounding the cerebral aqueduct (Fig. 8A,B), there were no dramatic differences in the appearance of astrocytes at 5 days of age.

Qualitative histologic examination of the 12d animals revealed astrocytes throughout all cortical layers (Fig. 2). When sections were graded for the relative abundance of GFAP positively stained cells, there was a 1.9 × (*p *< 0.05) increase in cellular amount in the 12d hydrocephalic animals when compared to the 12d control animals. Examination of the tissue sections, in the periventricular white matter (Fig. 7C,D) and the cerebral aqueduct (Fig. 8C,D) showed no obvious differences between hydrocephalic and control rats.

In the 21d hydrocephalic animals, the group with the most severe form of hydrocephalus, GFAP stained cells were distributed evenly throughout all cortical layers whereas in the 21d control animals, they were within cortical layers 1 and 2 (Fig. 2). When tissue sections were graded, there was a significant 1.97x increase in the relative number of astrocytes in the hydrocephalic animals when compared to age matched controls (*p *< 0.05) (Fig. 6). Additionally, a large concentration of GFAP positive cells was found to be present in the peri-ventricular white matter (Fig. 7E,F), and in the cerebral aqueduct (Fig. 8E,F).

At 36d, the hydrocephalic animals also had an increase in GFAP labeled astrocytes throughout all cortical layers when compared to the 36d control animals (Fig. 2). After grading, a 1.46 × increase in astrocytes was found in the hydrocephalic animals when compared to their control counter parts (*p *< 0.05). Additionally, GFAP labeled cells were more abundant in both the periventricular white matter (Fig. 7H,I) and the area surrounding the cerebral aqueduct (Fig. 8H,I).

### Astrocyte histology: treated hydrocephalus

Following shunting, the distribution of astrocytes was altered in both the 21d and 36d animals. In the 21d shunted animals, a marginal decrease in GFAP labeled astrocytes was observed throughout all layers of the brain tissue (Fig. 2). Furthermore, a non-significant decrease of 18.6% was measured after grading (Fig. 6). In the periventricular white matter (Fig. 7G) and the area surrounding the cerebral aqueduct (Fig. 8G), astrocyte density was slightly reduced in the shunted animals when compared to their hydrocephalic counterparts.

In the 36d shunted animals, there was a noticeable decrease in relative abundance of GFAP labeled astrocytes in the cortical layers (Fig. 2). After grading, there was a significant 31.5% decrease (*p *< 0.05) in the relative number of astrocytes when compared to age-matched untreated hydrocephalic animals, and the grade was close to that of the control rats. Examination of the periventricular white matter (Fig. 7J) and the area surrounding the cerebral aqueduct (Fig. 8J), showed a more dramatic reduction in the numbers of labeled astrocytes in the shunted group when compared to the untreated 36d hydrocephalic littermates.

### Microglia histology: untreated hydrocephalus

Although the microglial cells were not quantified, the number of ILB4 positive cells in the cortical sections did not increase or decrease dramatically with developmental age or the severity of hydrocephalus. However, the cellular morphology of the microglia was noticeably different (Fig. 3). This change toward activated microglia has been well documented as a response to injury [39,40,44]. In activated microglia, processes become shorter and thicker, while cell bodies and cellular processes stain more intensely. Hydrocephalic animals in all age groups appeared to have at least some activated microglia with a thicker cell body giving off shorter and thicker branches and cellular processes.

Normal differentiating microglia also change morphology. The typical maturation process of a microglial cell is demonstrated beautifully by Orlowski *et al *[42], and was used for maturation comparison in the current study (Fig. 1). In his paper, Orlowski described immature microglia as having an amoeboid shape with little cellular differentiation. As the microglial cell matured, cellular processes became more pronounced, and by postnatal day 30, the cell was fully ramified with extensively branched and long, fine processes. ILB4-immunostained cells in both control and hydrocephalic animals at the different time-points clearly exhibited the changes in microglial morphology with maturation (Fig. 3 left column). In 5d control animals, the majority of microglia had an amoeboid shape, with very few cells having the beginning stages of cellular processes (Fig. 3, top row). The hydrocephalic 5d animals had microglial cells that looked similar to the postnatal day 0 animals from Orlowski's figure (Fig. 1 top) and there was a small increase in the relative number of these amoeboid-like cells especially in the peri-ventricular white matter, although cell counting was not performed. In 12d control animals, the normal microglial population was more differentiated with established processes, although the processes were not fully ramified as in mature microglial cells (Figs. 1, 3, 2^nd ^row), while microglia in the hydrocephalic 12d animals had shorter processes, and their cell bodies appeared to be thicker than those in the control animals (Fig. 3, 2^nd ^row). By 21d, the microglia in control animals had reached their fully ramified state, with branched processes that were long and slender (Fig. 1, 3, 3^rd ^row). Microglia in the 21d hydrocephalic animals had altered and activated morphology with a wider cell body and shorter, thicker processes (Fig. 3, 3^rd ^row). These changes in microglia morphology toward that of an activated state continued in the 36d hydrocephalic animals (Fig. 3, 4^th ^row). There were no dramatic alterations in the microglial populations of the periventricular white matter (Fig. 7) or the cerebral aqueduct (Fig. 8) in any of these untreated animal groups.

### Microglia histology: treated hydrocephalus

Histologic examination of ILB4 stained sections shows that shunting altered the microglial morphology from the appearance of microglia in the untreated brain. Following shunting (both long and short term), the shape of the microglia began to return towards that of a resting microglia cell. Microglia in shunted animals had thicker cell processes than those of resting microglia, but these processes were not as broad and aggravated as the activated microglia in the untreated hydrocephalic animals (Fig. 3, 3^rd ^and 4^th ^rows). The microglial cell activation in the 36d shunted animals was almost completely reversed, with less intense staining and a return of fine cellular processes. Additionally, there was a small increase in number of microglia cells in the periventricular area (Fig. 7). Shunting was also found to increase the overall number of microglial cells present in the peri-aqueductal area for both shunt durations, and appeared as small, amoeboid shaped cells on the ventricular surface (Fig. 8G,J).

## Discussion

Gliosis is the brain's natural response to injury [45]. In hydrocephalus, the stretch and compression of the brain tissue caused by the enlarged ventricles can instigate the proliferation of astrocytes and microglia. This stretch and compression can also cause damage to connectivity pathways, interrupt cellular metabolism, cause cellular death or dysfunction and impede cerebral blood flow [1-3,20,46-48]. Increased numbers of glial cells can inhibit neurite outgrowth, and impede recovery of the brain tissue [49-51]. This impaired recovery, along with the cell death originally caused by hydrocephalus, may contribute to the neurologic deficits experienced by many patients [13-19].

Several different animal models are available to study hydrocephalus, and one widely used method is the induction of obstructive hydrocephalus by a mechanical blockage of CSF flow pathways with Kaolin, causing closure of the fourth ventricle outlets. However, these injections induce rapid onset hydrocephalus that can be variable depending on the location of the CSF obstruction and this method cannot mimic all types of hydrocephalus. Although not perfectly mimicking the human form of hydrocephalus, we utilized the naturally occurring H-Tx model of rodent congenital hydrocephalus for our studies. These animals develop hydrocephalus due to an alteration of the cerebral aqueduct, which occurs between embryonic day 18 and post-natal day 5 [30-36]. Although intracranial pressure in these animals does not increase until postnatal day 10 [52], ventriculomegaly develops steadily and progresses until the animals develop a severe state of hydrocephalus by approximately postnatal day 15. If these animals are left untreated, the hydrocephalic H-Tx rats will usually die by 4–6 weeks of age, with only a few surviving longer [52].

By utilizing a naturally occurring model of hydrocephalus, we have demonstrated that reactive astrocytosis, as discerned by Western blots of GFAP and GFAP-immunohistochemistry, increase in parallel with the onset and progression of hydrocephalus. Increases in GFAP levels and relative astrocyte number were detected as early as 5d of age, and although there was also an age dependent increase in the overall astrocytosis with development [53,54], the largest astrocytic responses were found in 21d and 36d hydrocephalic animals. These dramatic astroglial increases occurred as the hydrocephalic condition advanced to a more severe state, indicating a relationship between the severity of hydrocephalus and the amount of astrogliosis.

Shunting hydrocephalic animals and allowing them to survive to 21d (6d post- shunt) or 36d of age (21d post shunt) was effective in reducing reactive astrocytosis; GFAP levels and the number of GFAP-positive cells were decreased, and cortical thickness was restored towards control levels. Shunting for as little as 6d was effective in reducing the amount of GFAP present in both the parietal and occipital cortices, with the reduction being more dramatic in the occipital cortex. This difference may be because the occipital cortex is more severely affected by the hydrocephalic condition both grossly and in amount of GFAP present, so treatment could have a more dramatic result proportionally. The longer three-week shunt duration also provided a dramatic reduction in the level of GFAP present in both the parietal cortex and the occipital cortices. Although this reduction was significant, it was not as pronounced in occipital cortex of the 36d animals as it was in the occipital cortex of the 21d animals. One possible reason for this reduced effect could be due to partial occlusion of the shunt with time by growth of connective tissue into the distal end of the shunt catheter. Although CSF flow was observed in all shunts at the time of sacrifice, this potential growth may reduce the efficacy of the shunt and prompt re-activation of glial cells. Obstruction of the shunt is a common problem in the clinical setting, and many children undergo revisions to correct this [55,56], therefore it is not unlikely that the rats may suffer from this same complication.

Histologic examination of GFAP labeled astrocytes revealed increases in relative number of positively stained cells, which correlated with the increasing GFAP levels detected in the Western blot analysis. The increased staining in periventricular regions, notably the periventricular white matter and the periaqueductal gray, suggests that the stretch and compression that accompany ventriculomegaly could be a primary injury mechanism.

Following shunting, the relative number of astrocytes was altered in both the 21d and 36d animals. In both of the shunted groups, astrocytes were present in the peri-ventricular area but were only found in a narrow band just outside the ventricles without extending far into the cortex. Additionally, in the area of the third ventricle leading to the aqueduct and in the cortex, the density of astrocytes was greatly reduced. These reductions are most likely due to the effective diversion of excess CSF to other sites of absorption, which keeps intracranial pressure levels under control and reduces the amount of stretch and compression on the cortex.

Hydrocephalus also had a marked effect on microglial morphology. In hydrocephalic animals of all ages, with microglia at various developmental stages, these cells responded to the hydrocephalic condition. Activation of immature microglia in the younger 5d and 12d animals consisted of less ramified and smaller processes, which made the cells appear as though they were developmentally delayed when compared to their control counterparts. This is supported by the observation that the broad, shortened appearance of an activated microglia in 12d hydrocephalic animals is similar to the 8d developing microglia shown by Orlowski *et al*. (Fig. 1) [42]. It is possible that hydrocephalus is causing the morphologic change of these microglia through a modified activation in these young cells, or it may be an actual delay in microglial developmental caused by the presence of hydrocephalus. The older 21d and 36d hydrocephalic animals underwent a more classical transformation into an activated microglial cell. As the microglia cells become activated, they change their morphologic shape and function from that of a resting supportive cell to that of a macrophage cell that helps to rid the brain of damaged tissue [40,57].

Shunting the hydrocephalic animals altered the microglia morphology in both the long and short term survival groups, such that they progressed towards a typical resting state. This effect was most dramatic in the 36d shunted animals, where the microglia had regained most of their fine cellular processes, their staining intensity had returned to normal, and the relative number of cellular branches returned to that of their control counterparts. Together, these data imply that shunting reversed the microglial activation. Research has also shown that following the insertion of a shunt, other distortions of the brain occurring due to hydrocephalus begin to revert back to the control state. A few examples of these other reversals include the decrease in ventricular volume, increase in cortical thickness, the increase in number of cortical laminae, and improvement of cortical connections [58-60]. Furthermore, some researchers believe that reducing the presence of the glial scar can aid in the recovery of damaged tissue by forming a barrier [61], and that modulation of the glial response may actually be used to help promote CNS repair [62,63]. Therefore understanding and controlling the glial response in hydrocephalus may be helpful in reducing rigidity of the brain due to hydrocephalus [64].

**Figure 9:**
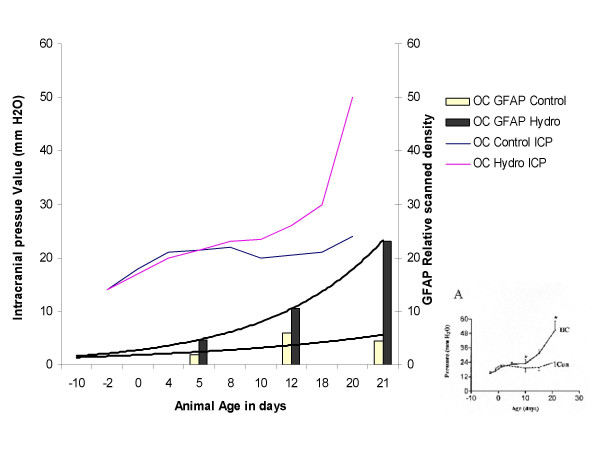
Correlation between intracranial pressure and astrogliosis. Representation of intracranial pressure data from H-Tx rats by Jones *et al*. [52] plotted with GFAP levels in the occipital cortex from this study, over time. Original graph of ICP from [52] is in the lower right corner. In the overlay graph, the similarity is shown between the large increase in GFAP levels occurring in the occipital cortex of the 21d hydrocephalic animals and the increase in ICP in similar animals. The fit of the GFAP regression line in the hydrocephalic animals was 0.9997 and the control animals 0.5128. This correlation between the hydrocephalic GFAP levels and the increase in ICP may imply a causative effect on increasing gliosis.

Although gliosis in these animals was initiated at an early time-point during hydrocephalus, the severity did not escalate until the animals were older. Jones *et al*. previously reported that intracranial pressure in hydrocephalic H-Tx rats increases around 10d of age [32,65,66], this correlates with the overall increase in GFAP expression (Fig. 9) and the amount of GFAP stained cells that were seen in the hydrocephalic animals in this study. Twelve days is the age when microglia morphology began to change dramatically, from a resting state to the activated state of a scavenging cell. One can hypothesize that a possible stimulus for this dramatic increase in gliosis occurring during this transition state of hydrocephalus could therefore be an increase in ICP. Further support for this mechanism includes evidence following shunting when presumably ICP decreases as it does in humans, GFAP levels fall, cellular proliferation of astrocytes decreases, and microglial morphology returns to normal.

## Conclusion

This research has shown that, contrary to the previously held belief that gliosis in the hydrocephalic brain is restricted only to the peri-ventricular white-matter [8,20,65,67-70], gliosis extends through all of the cortex and the peri-aqueductal area. A correlation between the increase of ICP due to hydrocephalus and the onset of gliosis has been demonstrated and therefore it is possible that the increase in intracranial pressure may be one of the triggers for the onset of gliosis. The implantation of a shunt, either short or long term, was effective at reducing the increase in GFAP due to hydrocephalus, and led to a reduction in the overall presence of both astrocytes and microglia. Through appropriately timed shunting, this gliosis can be prevented from increasing and controlled at levels closer to that of the control rats. By understanding the timing and progression of gliosis, it is now possible to investigate the appropriate use of glial inhibitors and other neuroprotective agents to further control the process of reactive gliosis, and to reduce the detrimental effects that gliosis can impart on the brain.

## Competing interests

The author(s) declare that they have no competing interests.

## Authors' contributions

JMM participated in the design of the study, carried out all surgeries and technical procedures, analyzed data, performed statistical analysis and composed the manuscript. JPM participated in design of the study, acquired all funding and critically evaluated and aided in the revision of the manuscript. All authors have read and approved the final manuscript.
